# Greenhouse Gas Emissions and Cost Trade-Offs of Renewable Feedstocks for Methyl Methacrylate Production

**DOI:** 10.1021/acssuschemeng.6c03784

**Published:** 2026-06-02

**Authors:** Sarah Bowler, William C. Hunt, Jon McKechnie

**Affiliations:** Low Carbon Energy and Resources Technologies Research Group, Faculty of Engineering, 6123 University of Nottingham, Nottingham NG7 2RD, U.K.

**Keywords:** greenhouse gas emissions, raw material cost, biomass, direct air capture, methyl methacrylate, green premium, social cost of carbon

## Abstract

Replacing fossil feedstocks with renewable, drop-in intermediates offers a rapid defossilization strategy while exploiting existing infrastructure. This study quantifies the cradle-to-gate greenhouse gas emissions and raw material costs for methyl methacrylate (MMA) via a representative ethylene (“C2”) route using fossil, biomass, and direct-air-capture CO_2_ feedstocks. Results suggest that conventional MMA emits 3.43 kg CO_2_-eq kg^–1^. Switching to biomass or CO_2_ feedstocks yields negative emissions of −0.79 and −1.13 kg CO_2_-eq kg^–1^, respectively. Uncertainty analysis demonstrates −0.88 to −0.40 kg CO_2_-eq kg^–1^ (biomass) and −1.23 to −1.03 kg CO_2_-eq kg^–1^ (CO_2_). Raw-material costs rise from $0.65 kg^–1^ (fossil) to $1.52 kg^–1^ (biomass) and $2.66 kg^–1^ (CO_2_), driven by renewable ethylene and formaldehyde. The green premium required for cost parity with fossil fuel-based MMA is $0.31–1.04 kg^–1^ (biomass) and $1.27–2.12 kg^–1^ (CO_2_), equivalent to a 16–52% and 63–106% markup. Propagated to consumer products, the highest premium inflates prices by <15% for an acrylic sheet and <1% for higher-value items (rear car lamps; LCD televisions). Eliminating the premium requires carbon prices of $72–296 tn^–1^ CO_2_ (biomass) and $269–512 tn^–1^ CO_2_ (CO_2_). These results position biomass drop-ins as a near-term strategy, while CO_2_-derived options need further improvements to reach cost parity.

## Graphical abstract

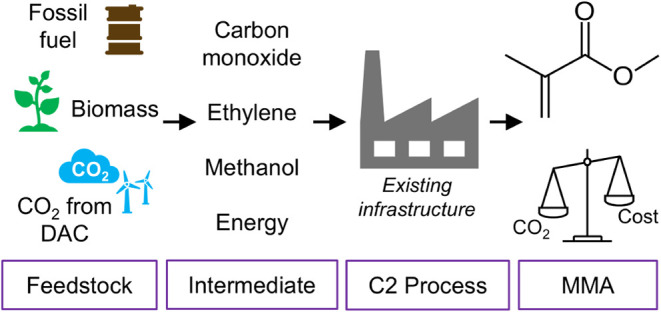

## Introduction

The current organic chemical industry relies on key building blocks, namely, ammonia, methanol, light olefins, and aromatics.^1^ Renewable production of these key intermediates as “drop-in” chemicals would, therefore, have a dramatic effect on the industry’s overall carbon emissions. Furthermore, infrastructure already exists to process these building blocks,^2^ lowering capital requirements and leveraging decades of process optimization and development. This approach also offers the opportunity to simultaneously coprocess a mix of biogenic and fossil-fuel feedstocks, a practice already adopted for biofuel production.^3^ This flexibility in operation could help derisk renewable chemical production by facilitating feedstock switching based on pricing, policy, and market conditions.

Methyl Methacrylate (MMA) is the precursor for Poly Methyl Methacrylate (PMMA). PMMA has many favorable qualities, including being transparent, lightweight, and shatter- and scratch-resistant.^4^ The PMMA market is projected to grow from $5.1 billion in 2024 to $6.1 billion by 2029 due to increased demand from the construction, automotive, and electronics industries.^5^ This growing demand has motivated the industry to investigate and develop clean production technologies.

Renewable MMA production via fermentation routes to methacrylic acid (precursor to MMA) has been an area of focus for researchers and manufacturers for several years.^6^ Examples include a hybrid bio/chemo-catalytic route via citramalic acid with an overall glucose-to-methacrylic-acid yield of 65 mol %^7^ and itaconic acid routes reporting fermentation yields of 0.24–0.72 g g^–1^,^8^ followed by a 50% catalytic upgrading yield.^9^ However, all proposed fermentation routes originate from glucose, which has a maximum yield of 47.7 wt % when 1- mole of glucose reacts to form 1-mole of methacrylic acid, implying an inherent carbon loss and making high overall yields challenging. Even when assuming this theoretical yield, Lebeau et al.^6^ estimate that methacrylic acid production from glucose would be twice as costly as the commercial ethylene-based C2 Alpha process, making cost parity a significant challenge.

An alternative to fermentation is to use renewable chemical feedstocks in existing MMA infrastructure.^10^ The C2 Alpha feedstocks (methanol, ethylene, and CO) can all be produced renewably,^2^ making it a promising renewable production option. Recent estimates place the industry-average cradle-to-gate carbon footprint of MMA at 3.7 kg CO_2_-eq kg^–1^,^11^ while MMAtwo’s route-specific benchmarking reports 5.2–7.7 kg CO_2_-eq kg^–1^.^12^ These elevated figures reflect modeling without energy optimization, which partly explains the higher range but also raises questions about their representativeness of industrial practice. Adeoye et al. reported 8.74 kg CO_2_-eq kg^–1^ for C2 Alpha (Singapore) and 12.3 kg CO_2_-eq kg^–1^ for an in situ formaldehyde variant, driven largely by electricity for heating and cooling (68–87% of emissions), indicating sensitivity to energy supply and heat integration.^4^ Baseline footprints from MMAtwo rank technologies as C2 (ethylene-based) < C4 (isobutylene-based) < C3 (acetone-based), reinforcing the potential for low-carbon renewable MMA via the C2 Alpha process.

Despite the appeal of integrating renewable intermediates into the C2 Alpha process, no peer-reviewed study has quantified the associated greenhouse gas (GHG) emissions or costs. Here, we address this gap using a literature-based representation of the C2 Alpha process to compare MMA life cycle GHG emissions and costs across three U.S. feedstock scenarios: fossil fuel, biomass, and direct air capture (DAC) CO_2_. We also estimate the green premium and carbon price required for the renewable routes to achieve cost parity with fossil fuel-based production.

## Methodology

This study quantifies the cradle-to-gate life cycle GHG emissions and raw material cost of MMA via a representative C2 route, considering three alternative feedstocks: fossil fuel, biomass, and DAC CO_2_. It is intended as an academic, comparative screening assessment of prospective MMA production scenarios, rather than as a product carbon footprint declaration under ISO 14067. The purpose is to compare the relative greenhouse gas emission and raw material cost implications of these scenarios under a consistent, literature-based modeling framework.

- Fossil fuel route – Emission factors representing industrial fossil-fuel production processes and recent market prices were used to establish the range for GHG emissions and raw material production costs
- Renewable routes:
  - Biomass – Biomass resources were selected according to their appropriateness for chemical production, e.g., corn for ethylene (via ethanol dehydration) and woody biomass for methanol (via gasification and catalytic upgrading)
  - CO_2_ from DAC – Intermediates synthesized from CO_2_ captured from air using wind-derived renewable electricity and thermal energy

As both renewable routes rely on nascent technologies, GHG emission factors and costs were taken from academic literature. Uncertainty and sensitivity analyses was performed across the reported ranges, representing differences in modeling assumptions and scenarios.

The resulting cost ranges were compared to estimate the green premium required for renewable MMA to reach cost parity with fossil-fuel-based MMA. By combining the emission and cost ranges, the CO_2_ price required to bring biomass- and CO_2_-based MMA to cost parity with fossil fuel-based MMA was also calculated.

### Greenhouse-Gas Emissions and Raw Material Costing

The study’s primary aim is to integrate the decarbonization potential of different feedstock scenarios with cost implications for MMA production. Accordingly, GHG emissions and raw-material costs were evaluated per 1 kg_MMA_, with climate change assessed using the 100-year GWP, IPCC 2013. This focus on climate change impacts does not represent a complete assessment of environmental sustainability, and other impact categories should also be considered when assessing feedstock choice. Direct land-use change (dLUC) was treated on a feedstock-specific basis. Corn-based ethylene used literature data that included LUC, while no dLUC was assigned to woody biomass feedstocks (used for methanol, formaldehyde, and CO), assuming sourcing from existing managed, nonconverted woody biomass. This assumption is broadly consistent with US EPA RFS eligibility for planted trees and residues, and with GHG Protocol guidance attributing land-use change where product-driven expansion or conversion causes a carbon stock change.^13,14^ Indirect LUC and temporal forest-carbon dynamics were not modeled, so results represent attributional 100-year GWP estimates rather than dynamic forest carbon flows.

As the only difference between the three cases is the feedstock source, the cost was restricted to variable costs, including material and energy inputs. Capital investment, labor, and other operating costs were excluded, as these are assumed to be identical across feedstocks.

### System Boundary and Material Inventory

The production process (Figure 1) and material inventory (Table S1) were adapted from Adeoye et al.^4^ whose model is based on a series of Lucite International patents.^15,16^ The continuous injection of inert liquid CO_2_ (20 vol %) was removed from the inventory, as this would be internally recycled and the makeup was judged negligible. Energy requirements for methyl propionate and MMA production and purification follow Parvatker and Eckelman,^17^ using the minimum heating and cooling demands, reflecting a heat-integrated process. Heating and cooling requirements for methacrylic acid upgrading to MMA were taken from Moraru et al.^18^ The resulting inventory, therefore represents a literature-based representation of the C2 Alpha process. Although the adapted inventory is based on Lucite patents, patent-reported yields may not reflect optimized plant performance, and commercial plants likely achieve greater heat integration than captured by the stand-alone models used.

**Figure 1:**
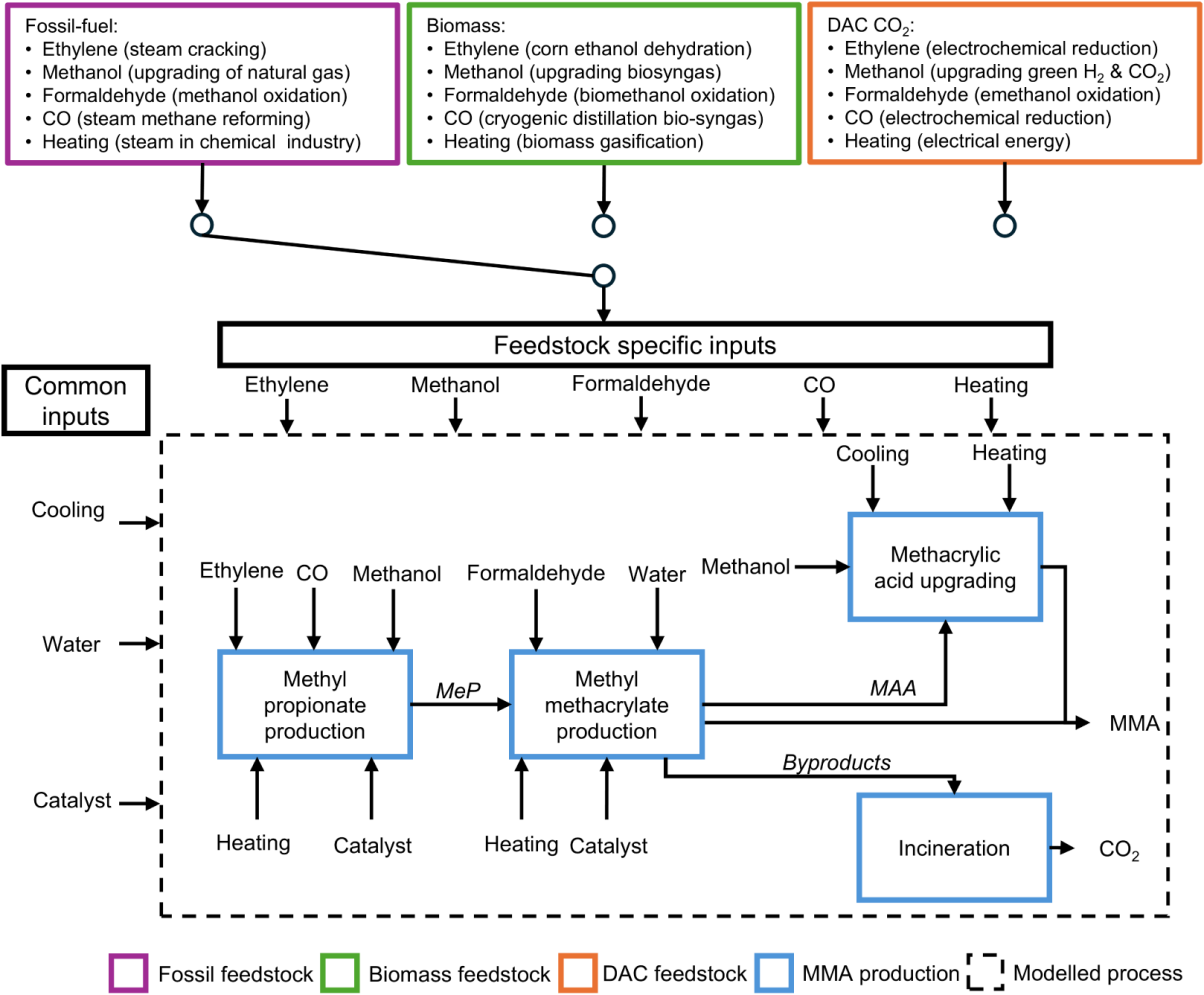
System boundary considered for MMA production via the C2 Alpha process. Upstream feedstock scenarios are taken from data representing industrial petrochemical processes (fossil fuel scenario) or recent literature data (biomass and DAC scenario). One feedstock scenario is considered at a time to give rise to the three comparative GHG emission and cost results.

A cradle-to-gate system boundary was used, as MMA is utilized in various industries and end products, consistent with existing studies.^4,11,12^ Renewable carbon was accounted for using the “–1/+1” approach, in line with common practice in Environmental Product Declarations (EN 15804 ^19^ and encouraged by stakeholders in the European biobased materials sector^20^). This approach assigns a negative value where renewable carbon enters the system (biomass growth or DAC) and a positive value when it leaves. On a cradle-to-gate basis, this yields negative emissions for renewable carbon retained in the product. Accordingly, direct emissions from byproduct combustion (Table S1) were counted only for the fossil route. For renewable intermediates, GHG emissions were adjusted to remove any storage credits already allocated, avoiding double counting.

The US plant location was considered, as it has the largest bioeconomy, with 637 of 1312 biorefineries globally located in the US.^21^ While no current US Alpha-MMA facility exists, a 350 kt yr^–1^ facility was previously planned.^22^ In addition, the developed models could be adapted to other locations by updating the material costs and emissions to the desired country.

### Data Collection

Emission factors and costs consistent between scenarios are presented in Table S2. For the fossil-fuel scenario, ecoinvent was used for the heating emission factor, with the remaining emission factors representing current U.S. fossil-fuel production routes. Chemical prices were taken from recent market reports and spot prices. For the two renewable scenarios, emission factors and costs were taken from peer-reviewed studies and are detailed below. All values, ranges, and sources are reported in Table S3.

Corn-based bioethylene emissions reported in the literature span 2.64–4.64 kg CO_2_-eq kg^–1^ ^24,25^ with several studies clustering around 3–4 kg CO_2_-eq kg^–1^.^26,27^ This study adopted 3.1 kg CO_2_-eq kg^–1^ ^28^ as the baseline for ethanol dehydration, with 2.64 kg CO_2_-eq kg^–1^ ^24^ and 4.64 kg CO_2_-eq kg^–1^ ^25^ used for sensitivity analysis. Costs for corn-based bioethylene range $1,300–2,300 tn^–1^ ^24,25^ in agreement with industry estimates.^29–31^ A baseline of $1,850 tn^–1^ was adopted, with $1,300–2,000 tn^–1^ as the range. Biomass methanol was represented by woody biomass gasification-to-syngas and catalytic synthesis. An emission factor range of 0.2–0.5 kg CO_2_-eq kg^–1^ was used, representing forestry residues and woody biomass,^32^ with a midpoint of 0.35 kg CO_2_-eq kg^–1^ used as the baseline. The IRENA report indicates biomass-methanol costs of $820–1,620 tn^–1^, falling to $250–630 tn^–1^ by 2050.^33^ Accordingly, the midpoint of $1,230 tn^–1^ was used as the baseline, with $250 tn^–1^ (low) and $1,620 tn^–1^ (high). Biomass formaldehyde emissions and costs were calculated by substituting scenario-specific methanol and electricity into the ecoinvent methanol-oxidation inventory.^23^ Biomass-derived CO was calculated using the biomass-to-syngas inventory from Bachmann et al.^34^ followed by cryogenic separation.^35^ Inputs were assigned emission factors for poplar wood from GREET,^36^ oxygen and water from ecoinvent,^23^ and biomass-derived electricity. The syngas footprint was calculated as 0.528 kg CO_2_-eq kg^–1^, comparable to 0.302 kg CO_2_-eq kg^–1^ reported by Cormos et al.,^37^ (excluding sequestration). Electricity requirements for the cryogenic distillation of CO are reportedly 52.8 and 106.4 kWh tn^–1^_CO_ ^38^ (within the reported industrial range^39^). Aggregating syngas and separation gives an emission factor of 0.347–0.609 kg CO_2_-eq kg^–1^_CO_ with the midpoint (0.478 kg CO_2_-eq kg^–1^_CO_) used as the baseline. Costs for bio-CO were adapted from the authors’ techno-economic analysis of biosyngas-to-butadiene.^40^ The capital and operating costs for syngas production were updated to 2023 prices, with additional capital added for cryogenic distillation.^38^ The resulting CO cost is $582 tn^–1^ (excluding H_2_ credit). Low and high costs were estimated from the cost variance for biomass-based methanol and ethylene. Biomass electricity was taken as 0.0488 kg CO_2_-eq kWh_e_^–1^ ^41^ and $0.081 kWh_e_^–1^.^42^ For thermal energy, emissions were adjusted using efficiencies (22.1% power plant vs ∼80% direct heat^43^), giving 0.0135 kg CO_2_-eq kWh_th_^–1^. Heat costs were estimated from woody biomass costs ($58.5 tn^–1^,^44^) plus inflation-adjusted transportation ($9.2 tn^–1^,^45^ ), yielding $0.0173 kWh_th_^–1^ at 80% efficiency.

Emissions for DAC–CO_2_ ethylene were calculated using the material inventory from two studies for a cascade/tandem system.^46,47^ The emission factor for CO_2_ was calculated using the GREET model for low-temperature adsorption^36^ with thermal energy supplied by renewable wind electricity. This resulted in emissions of 0.68–0.85 kg CO_2_-eq kg^–1^ with the midpoint (0.77 kg CO_2_-eq kg^–1^) used as the baseline. The ethylene price from Leonzio et al. using onshore wind electricity ($2.54 kg^–1^)^48^ was updated from a CO_2_ price of $40 tn^–1^ to a low ($100 tn^–1^), baseline ($230 tn^–1^), and high ($335 tn^–1^) price for DAC–CO_2_ based on the IEA’s future capture costs.^49^ This resulted in ethylene prices of $3009 tn^–1^ (low), $3824 tn^–1^ (baseline), and $4482 tn^–1^ (high). Methanol can be produced thermochemically from CO_2_ and H_2_ using a similar process to large-scale syngas-to-methanol production. Emissions for methanol from DAC–CO_2_ using wind electricity in the US are reportedly 1.01–1.57 kg CO_2_-eq kg^–1^.^50,51^ This study adopted 1.29 kg CO_2_-eq kg^–1^ as the baseline, with the range used as the lower and upper bounds. DAC-methanol costs were adapted from Huang et al.^52^ by updating the CO_2_ price to the aforementioned prices used for DAC-based ethylene, resulting in methanol prices of $930 tn^–1^ (low), $2054 tn^–1^ (baseline) and $2270 tn^–1^ (high). Similarly to biomass-based, DAC-formaldehyde was calculated by adapting the methanol oxidation ecoinvent inventory^23^ with scenario-specific inputs (electricity and methanol). DAC–CO emissions were calculated using the required CO_2_ (3.14 kg CO_2_ kg^–1^_CO_) and electricity (6.77 kWh kg^–1^_CO_, excluding electricity for hydrogen production) for electrochemical syngas.^53^ Aggregating the DAC–CO_2_ emissions used for ethylene with renewable wind electricity emissions resulted in CO emissions of 0.26 kg CO_2_-eq kg^–1^. Due to data scarcity, the range used for CO emissions was derived from the average variance for CO_2_-based methanol and ethylene. Similarly to methanol, the price for CO was calculated by updating the CO_2_ prices considered by Huang et al.^52^ resulting in costs of $230 tn^–1^ (low), $670 tn^–1^ (baseline), and $830 tn^–1^ (high). Wind electricity emissions were taken as 0.011 kg CO_2_-eq kWh^–1^ ^54^ and cost $0.030 kWh^–1^,^42^ with heat energy assumed equivalent.

### Uncertainty and Sensitivity Analysis

A stochastic uncertainty analysis was performed for all scenarios using triangular distributions for the emission factors and raw material costs (Table S3). For the DAC–CO_2_-based cases, the CO_2_ price input was varied consistently across all intermediates. As formaldehyde is produced from methanol, the stochastic uncertainty was linked to methanol’s. A single-point sensitivity analysis was also carried out for the two renewable routes to demonstrate the individual contribution of the intermediates.

### Economic Competitiveness

Two metrics were examined to determine the economic feasibility of biomass- or CO_2_-based MMA: the green premium (the surcharge required for renewable MMA) and the carbon price needed to eliminate that premium.

Raw material costs are only one component of the total cost of manufacturing MMA; operating expenses, depreciation, and profit margin must also be recovered in the sale price. To estimate these charges for the renewable feedstocks, the margin above the raw material cost of the fossil fuel scenario was calculated:

1 $$\mathrm{Margin}={\mathrm{Market price}}_{\mathrm{fossil}}-{\mathrm{Raw material cost}}_{\mathrm{fossil}}$$

This margin, which represents all nonmaterial costs plus profit, is assumed to remain unchanged when switching feedstocks. Accordingly, the market price of biomass- or DAC CO_2_-based MMA was obtained by adding the same margin to their material costs:

2 $${\mathrm{Market price}}_{\mathrm{renewable}}=\mathrm{Margin}+{\mathrm{Raw material cost}}_{\mathrm{renewable}}$$

The green premium is the difference between this price and recent market prices. This premium represents the extra cost that must be absorbed by the producer, accepted by the consumer, or offset by policy support.

3 $$\mathrm{Green premium}={\mathrm{Market price}}_{\mathrm{renewable}}-{\mathrm{Market price}}_{\mathrm{fossil}}$$

The required carbon price for renewable MMA to reach cost parity with fossil-based MMA was calculated by dividing the additional cost associated with renewable production by the emission reduction achieved by renewable MMA.

4 $$\frac{\$}{{\mathrm{tn}}_{\mathrm{CO}2}}=\frac{{\mathrm{Raw material cost}}_{\mathrm{renewable}}-{\mathrm{Raw material cost}}_{\mathrm{fossil}}}{{\mathrm{Emissions}}_{\mathrm{fossil}}-{\mathrm{Emissions}}_{\mathrm{renewable}}}$$

## Results and Discussion

### Feedstock Comparison

The life cycle GHG emissions for MMA production via the representative C2 Alpha process using fossil fuel, biomass, and CO_2_-based feedstocks are presented in Figure 2. While not a representation of commercial reality, the relative performance of fossil-based and renewable pathways is still relevant, as changes in the material inventory would affect all scenarios. Notably, both biomass- and CO_2_-based scenarios yield net negative cradle-to-gate emissions owing to atmospheric carbon embedded in the product. As end-of-life fate is not modeled, these results do not represent permanent carbon removal but instead reflect the cradle-to-gate difference between fossil and renewable feedstock scenarios under a common end-of-life assumption. DAC–CO_2_-based MMA has lower emissions than biomass, mainly because corn-derived ethylene has high upstream emissions, with >50% of emissions arising from farming inputs.^27^ Although second-generation, nonedible biomass feedstocks can exhibit lower GHG emissions, the more complex pretreatment and lower yields mean corn remains the dominant U.S. ethanol source.^28^ Unlike the renewable cases, fossil-based emissions are dominated by heating, driven by the energy intensity of methyl propionate production, the high emissions of industrial steam, and direct emissions from byproduct combustion.

**Figure 2:**
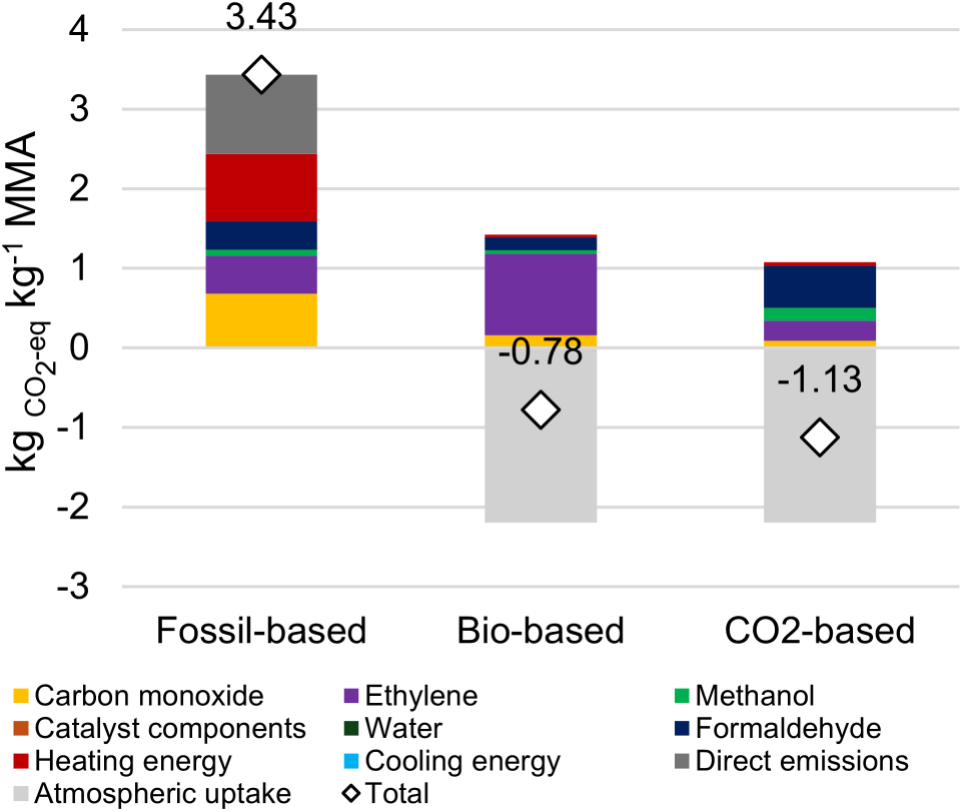
Comparative cradle-to-gate life cycle GHG emissions for fossil-based, biomass-based, and CO_2_-based MMA production via the Alpha process.

Figure 3 presents the raw material costs for the fossil fuel, biomass, and CO_2_-based MMA cases using the baseline costs. Both renewable cases are more costly than the fossil fuel-based route, with the biomass route being 2.3 times and the CO_2_ route being 4 times greater. As the primary raw materials for MMA production, ethylene and formaldehyde are the largest cost contributors to all three cases, contributing 30–48% and 32–40% of costs, respectively. CO shows the largest feedstock price variation, with biomass- and CO_2_-based routes costing 5.82 and 6.69 times more than the fossil-based case, respectively. However, because CO is used in small quantities, its effect on the final MMA cost is minor.

**Figure 3:**
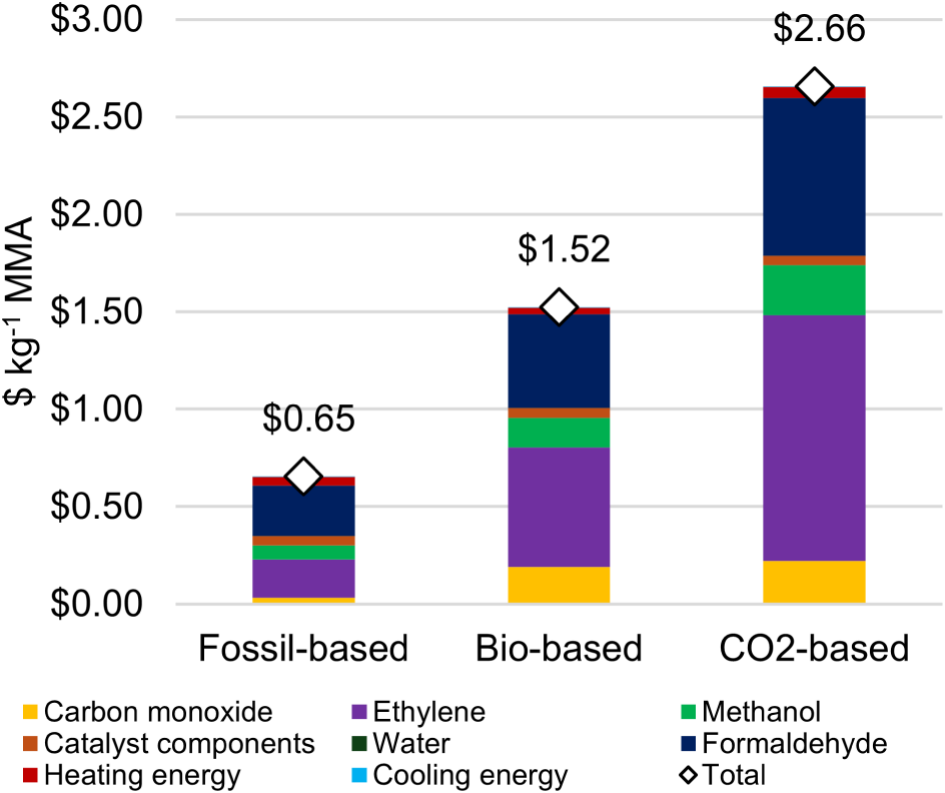
Comparative life cycle cost between fossil-based, biomass-based, and CO_2_ based MMA production via the Alpha process.

### Uncertainty and Sensitivity Analysis

Figure 4a presents the uncertainty analysis for the emission factor ranges in Table S3. The fifth–95th percentile ranges are 3.12–3.47, −0.88 to −0.40, and −1.23 to −1.03 kg CO_2_-eq kg^–1^ MMA for fossil-, biomass-, and DAC–CO_2_-based MMA, respectively. The lack of overlap indicates that, within the modeled uncertainty, fossil-based MMA consistently has the highest emissions, biomass-based MMA substantially reduces emissions, and DAC–CO_2_-based MMA has the lowest emissions. The single-point sensitivity analysis (Figure S1a–b) shows that biomass-based MMA emissions are most sensitive to ethylene, driven by the high-end estimate from McKechnie et al.^25^ (4.64 kg CO_2_-eq kg^–1^), which is based on the 2014 GREET model for bioethanol and Intratec data for dehydration to ethylene.^25^ By contrast, Benavides et al.^28^ used the 2022 GREET model and a literature-based dehydration model. This spread likely reflects differences in GREET version and assumed dehydration yield (54 wt %^25^ versus 57.5 wt %^28^). For the DAC–CO_2_ route, formaldehyde dominates emissions uncertainty because of its large contribution to the overall material inventory.

**Figure 4:**
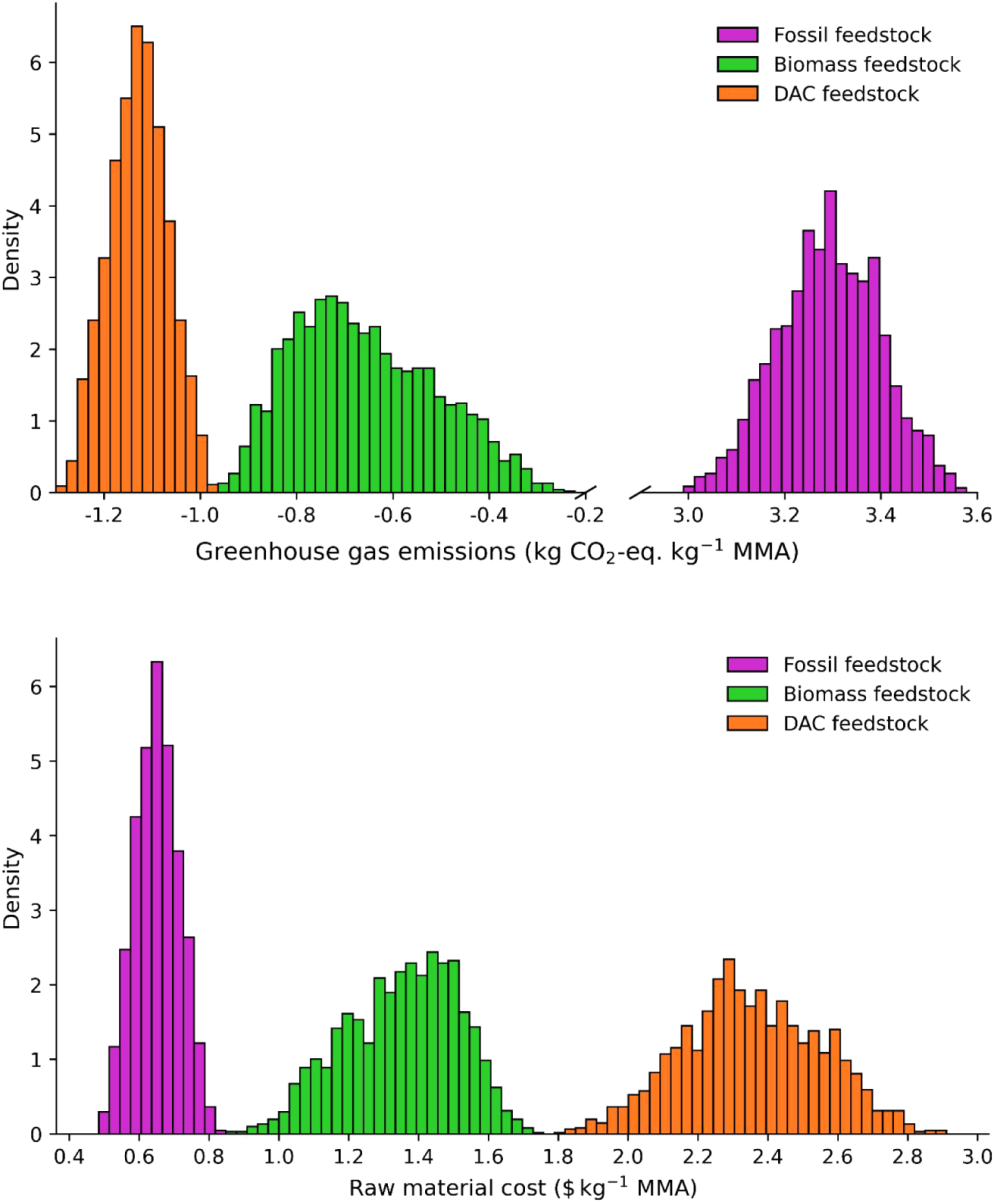
Uncertainty analysis for fossil-fuel, biomass-, and DAC–CO_2_- based MMA: a) Greenhouse gas emissions, b) Raw material costs.

Figure 4b shows the effect of raw material cost uncertainty (Table S3). In all cases, raw material cost follows the order fossil fuel < biomass < DAC–CO_2_, with fifth–95th percentile ranges of $0.55–0.76, $1.07–1.59, and $2.02–2.67 kg^–1^ MMA, respectively. The lack of overlap indicates that the cost ranking is insensitive to the modeled uncertainty. The single-point sensitivity analysis (Figure S1c–d) shows that biomass-based MMA cost is most sensitive to formaldehyde, primarily due to the quantity required rather than price volatility. For the DAC–CO_2_ route, ethylene has the greatest influence on cost, reflecting the early-stage nature of electrocatalytic CO_2_ reduction, where reported Faradaic efficiencies, cell voltages, and capital costs vary widely. Further discussion of the TRL of biomass- and CO_2_-based intermediates is provided in Supporting Information Section 4.

### Fossil-Based Comparison

The fossil-based Alpha process emissions for MMA are shown in Table 1. The calculated value is similar to the Plastics Europe estimate,^11^ this is expected because Plastic’s Europe represents an industry average across several production routes, while the C2 process is reported to be the lowest-emitting option.^12^ However, the calculated emissions are less than half of those reported by Adeoye et al.,^4^ largely because their study assumed electricity for heating and cooling, whereas this study uses steam and cooling water, both of which have lower GHG emission factors than grid electricity. Adeoye et al.^4^ also reported a much higher total heating and cooling demand (42.6 MJ kg^–1^ MMA versus 18.7 MJ kg^–1^ MMA here), whereas this study follows the minimum energy estimates reported by Moraru et al.^18^. These results suggest Adeoye et al.^4^ modeled a process without heat integration, which is unlikely to represent the commercialized process.

**Table 1:** Comparative Emissions and Costs for Fossil-Based MMA Production via the Alpha Process

Source	Value	Reference
**Emission factor (** **kg CO_2_-eq** **kg** ^**–1**^ **)**		
Virgin C2	5.28	^12^
Alpha Lucite process (AL-MMA)	8.74	^4^
Industry average	3.70	^11^
Literature based Alpha process	3.43	This work
**Cost ($ tn** ^**–1**^)		
North America Q1 2025	1970	^55^
North America June 2024	1980	^56^
US, May 2019–2020	2870–1450	^57^
US 2023 average	2000	^58^
Raw material costs	670	This work

Representative MMA market prices were collected from multiple online sources (Table 1). The fossil-derived MMA cost calculated in this work ($670 tn^–1^) is a raw material cost estimate, calculated by combining the literature-based Alpha process material balance with chemical prices from market reports and spot prices (Table S3). As this estimate excludes labor, utilities, maintenance, overheads, depreciation, and return on capital, it is expected to fall below market prices. Market volatility can further widen or narrow this difference, evidenced by the range of values presented for MMA.

### Reaching Cost Parity

The uncertainty analyses for biomass- and CO_2_-based MMA suggest a consistently higher material cost for MMA. This section explores the green premium or carbon tax required for renewable MMA to be competitive with fossil fuels.

Using a MMA wholesale price of $2.00 kg^–1^, the fossil route exhibits a raw-material cost of $0.65 kg^–1^ ($0.5–0.76 kg^–1^) and hence a manufacturing margin range of $1.24–1.45 kg^–1^ (eq 1). Holding this manufacturing margin range constant:

- Biomass-based MMA requires a selling price of $2.31–3.04 kg^–1^, a green premium of $0.31–1.04 kg^–1^ or +16–52% increase
- CO_2_-based MMA selling price is $3.27–4.12 kg^–1^, implying a premium of $1.27–2.12 kg^–1^ or a +63–106% price mark-up

Three examples are used to illustrate the impact on consumer products using the highest MMA price of $4.12 kg^–1^, as presented in Table 2. Even this worst-case premium adds <15% to the price of an acrylic sheet and <1% to higher-value manufactured goods, illustrating that downstream processing, distribution, and retail margins dilute increased MMA cost impacts.

**Table 2:** Cost Contribution of MMA in Consumer Products

Product	Current retail price	PMMA share of product mass	Baseline MMA share of price^a^	Retail price with highest MMA premium^b^	Change
2 mm clear cast acrylic sheet	17.16 ^59^	100%	11.7%	19.29	12%
Passenger-car rear lamp	100 ^60^	27%^61^	0.5%	100.57	0.6%
20-in. LCD television	170 ^62^	22%^63^	0.3%	170.46	0.3%

a Assumes $2 kg^–1^ MMA.

b Assumes $4.12 kg^–1^ MMA (upper end of all renewable cases).

An alternative to a green premium, which puts the price increase on the consumer, is to apply a price to the carbon reduction achieved and paid to/by the producer to achieve cost parity. Using the range of emissions and costs presented in Figure 4 the carbon price required to reach cost parity with fossil fuels are $72–296 tn^–1^ CO_2_ and $269–513 tn^–1^ CO_2_ for the biomass-based and CO_2_-based routes, respectively (Figure 5). The reported 2020–2050 Social Cost of Carbon (SCC) ranges from $190 to 310 tn^–1^ CO_2_ using a 2% Near-term Ramsey Discount Rate.^64^ Notably, the biomass-based MMA pathway falls within or below this range, suggesting biomass-derived intermediates could be a viable near-term route to renewable MMA. This is largely because the pathway relies on bulk platform molecules (methanol, CO, ethylene) whose biomass-based production has already been demonstrated (see Supporting Information, Section 4 for details on the TRL of biomass- and CO_2_-based intermediates). However, this reflects GHG-emission and cost competitiveness only, and broader feedstock selection would require assessment of additional environmental and resource indicators, including land use, water demand, biodiversity impacts, and competition for biomass resources.

**Figure 5:**
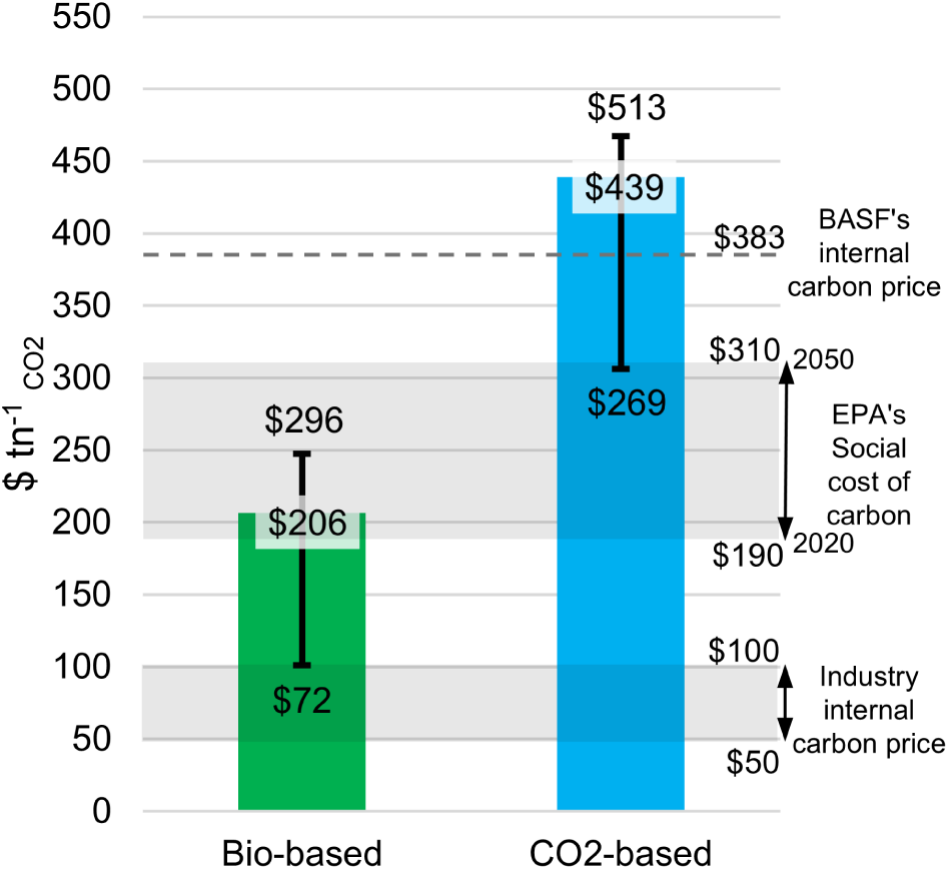
Cost of carbon for each route to reach cost parity with fossil fuel-based MMA production. Gray regions represent the EPA’s SCC for 2020–2050 using the 2% Near-term Ramsey Discount Rate and industry’s median internal carbon price range.

Between 2019 and 2022, the share of chemical firms using internal carbon pricing in investment decisions rose by 67%.^65^ BASF reportedly applies €340 tn^–1^ CO_2_ (≈$383 tn^–1^ CO_2_), above the 2050 SCC, whereas surveys report a 2023 median of ∼$50–100 tn^–1^ CO_2_, below the current SCC.^66^ This lower median highlights the disparity between the perceived monetary value of carbon emissions by industry. Nevertheless, the biomass-based MMA pathway falls near the range many firms use in capital budgeting, supporting biomass-derived drop-ins in existing chemical infrastructure.

No published studies of renewable MMA production report comparable carbon-pricing requirements. However, technoeconomic and life cycle assessments exist for acrylic acid, another monomer produced at megatonne scale with a comparable price point to MMA ($1.40–1.65 kg^–1^ ^67^). The renewable route to acrylic acid is via either (i) a bespoke fermentation to produce a renewable intermediate followed by catalytic upgrading^67^ or (ii) a novel catalytic process to upgrade glycerol.^68^ Existing studies suggest carbon prices of $269–500 tn^–1^ CO_2_ are needed to reach parity with fossil-fuel-based acrylic acid.^67,68^ This is higher than the biomass-based MMA route presented here and reinforces the benefit of leveraging existing infrastructure and commercially mature process technologies rather than bespoke processes for renewable chemical production.

## Conclusion

An approach to defossilizing chemical production is the use of renewable drop-in intermediates in existing petrochemical assets. This work calculated cradle-to-gate life cycle GHG emissions and raw material costs of fossil-based, biomass-based, and CO_2_-based MMA via a literature-based representation of the C_2_ Alpha process. Fossil-based MMA demonstrated emissions of 3.43 kg CO_2_-eq kg^–1^. Both renewable routes yielded lower emissions using the raw material emission factors and ranges collected from literature, with biomass-based MMA yielding −0.79 (−0.88 to −0.40) kg CO_2_-eq kg^–1^ and CO_2_-based MMA being −1.13 (−1.23 to −1.03) kg CO_2_-eq kg^–1^. Despite being lower in emissions, both renewable feedstock options were higher in cost than the fossil-based route $0.65 kg^–1^ ($0.5–0.76 kg^–1^). Biomass-based MMA demonstrated a cost of $1.52 ($1.07–1.59) kg^–1^ while CO_2_-based MMA was $2.66 (2.34–2.67) kg^–1^. The selling price required for biomass-based MMA to be economically viable was calculated as $2.31–3.04 kg^–1^ (16–52% mark-up), CO_2_-based MMA required a selling price of $3.27–4.12 kg^–1^ (63–106% mark-up). The carbon price required for the renewable routes to be cost-competitive with current market pricing was $72–296 tn^–1^CO_2_ and $269–513 tn^–1^CO_2_ for the biomass-based and CO_2_-based routes, respectively. The biomass-based MMA range is near current industrial carbon costs, reinforcing the value of leveraging existing technologies and infrastructure. In contrast, CO_2_-based routes exceed present ranges, indicating the need for further technological advancement. Biomass-based MMA may therefore represent a nearer-term route to cost-competitive defossilization under the assumptions considered. However, feedstock choice should not be determined by GHG emissions and carbon-price parity alone. Future work should extend the assessment to additional environmental and resource indicators, including land use, water demand, eutrophication, acidification, biodiversity impacts, feedstock availability, and competing uses.

## Supplementary Material



## Data Availability

The data underlying this study are available in the published article and its Supporting Information.

## Notes

Material inventory, summary tables containing emission factors and costs, single point sensitivity analysis, and discussion on current TRL levels of biomass and CO_2_-based intermediates (PDF)
