# Supplementary material for: Greenhouse Gas Emissions and Cost Trade-Offs of Renewable Feedstocks for Methyl Methacrylate Production
Source: ACS Sustain Chem Eng. 2026 Jun 2;14(23):10839–47. doi: 10.1021/acssuschemeng.6c03784 (PMC13273798; doi:10.1021/acssuschemeng.6c03784)
Supplement: Supplementary file 1 [file sc6c03784_si_001.pdf]

Supplemental Information

Greenhouse Gas Emission and Cost Trade-offs of Renewable Feedstocks for Methyl Methacrylate Production

Sarah Bowler[†]*, William C. Hunt[†], Jon McKechnie[†]

[†] Low Carbon Energy and Resources Technologies Research Group, Faculty of Engineering, University of Nottingham, Nottingham, NG7 2RD, UK

***Corresponding author. E-mail address: sarah.bowler@nottingham.ac.uk**

Number of Pages – 7

Number of Tables – 3

Number of Figures – 1

Table of contents: **Page no.**

Section 1: Material Inventory **S1**

Table S1: Inventory for MMA produced via the Alpha process. Material use adapted from Adeoye et al. (1). Energy use from Parvatker & Eckelman (2) and Moraru et al. (3).

Section 2: Emission factor and cost summary **S2**

Table S2: Emission factors and costs used for fossil, biomass, and CO_2-based MMA

Table S3: Emission factor and chemical price ranges used for biomass and CO_2-based MMA

Section 3: Single point sensitivity analysis results **S3**

Figure S1: Single-factor sensitivity of the emission factors for MMA (a) biomass-derived and (b) CO_2-derived intermediates and raw-material prices for MMA (c) biomass-derived and (d) CO_2-derived intermediates. Blue bars: low-end cost estimates; orange bars: high-end estimates

Section 4: Technology readiness level of biomass and CO_2-based chemicals **S4**

References **S5**

Section 1: Material Inventory

Table S1: Inventory for MMA produced via the Alpha process. Material use adapted from Adeoye et al. (1). Energy use from Parvatker & Eckelman (2) and Moraru et al. (3).

Material	Quantity per kg MMA
Ethylene (kg)	3.30E-01
Methanol (kg)	1.24E-01
Formaldehyde (kg)	3.75E-01
Carbon monoxide (kg)	3.30E-01
Palladium catalyst (kg)	1.24E-10
Silica sand (kg)	7.63E-04
Caesium (kg)	5.76E-05
Zirconia (kg)	7.70E-06
Water (kg)	3.35E-02
Heating energy (kWh)	1.95E+00
Cooling energy (kWh)	1.68E+01
Direct emissions (kg) *(combustion, fossil only)*	9.86E-01
Sequestered carbon (kg)	-2.20E+00

Section 2: Data collection

Table S2: Emission factors and costs used for fossil, biomass, and CO_2-based MMA

Material	Emission Factor (kg CO_2-eq kg^{-1})	Cost ($ kg^{-1})	Comments
Cooling water	Water production, deionised (RoW)	Cooling water (US)	Proprietary
Palladium catalyst	6.33[(4)]	250[(5)]	Pt/Gamma Al2O4 catalyst used as surrogate
Silica sand	0.02[(4)]	53[(6)]	
Zirconia	10.49[(4)]	21.84[(7)]	Zirconium used as surrogate for emissions
Caesium	Unavailable	145.06[(8)]	Adjusted from caesium carbonate
Water	Water production, deionised (RoW)	Process water (US)	Proprietary

Table S3: Emission factor and chemical price ranges used for biomass and CO_2-based MMA

Material	Emission factors (kg CO_2-eq kg^{-1})			Cost ($ tn^{-1})		
	Low	Base	High	Low	Base	High
**Fossil fuel-based**						
Ethylene	0.90[(9)]	1.10[(9)]	1.70[(9)]	415[(10)]	592[(10)]	738[(10)]
Methanol	0.50[(11)]	0.58[(11)]	0.65[(11)]	300[(12)]	580[(12,13)]	860[(13)]
Formaldehyde	0.75[(11)]	0.90[(11)]	1.05[(11)]	400[(14)]	694[1]	987[1]
CO	1.24[(15)]	-[2]	2.49[(15)]	50[(16)]	100[(16)]	150[(16)]
**Biomass-based**						
Ethylene	2.64[(17)]	3.10[(9)]	4.64[(18)]	1300[(17)]	1850[(19)]	2000[(17)]
Methanol	0.20[(20)]	0.35[(20)]	0.50[(20)]	250[(21)]	1230[(21)]	1620[(21)]
Formaldehyde	0.28[(22)]	0.43[(22)]	0.59[(22)]	262[(22,23)]	1290[(22,23)]	1699[(22,23)]
CO	0.32[(24,25,26)]	0.45[(24,25,26)]	0.57[(24,25,26)]	264[3]	582[(26,27)]	698[3]
**CO_2-based**						
Ethylene	0.68[(22,28)]	0.77[(22,28,29)]	0.85[(22,29)]	3009[(30, 31)]	3824[(30, 31)]	4482[(30, 31)]
Methanol	1.01[(32)]	1.29[(32,33)]	1.57[(33)]	928[(30,34)]	2054[(30,34)]	2272[(30,34)]
Formaldehyde	1.12[(22)]	1.41[(22)]	1.71[(22)]	977[(22,23)]	2158[(22,23)]	2387[(22,23)]
CO	0.21[4]	0.26[(4,35)]	0.30[4]	282[(30,34)]	669[(30,34)]	828[(30,34)]

[1] Base and high values calculated based on relationship of low formaldehyde and methanol price
[2] Proprietary, ecoinvent value.
[3] Low and high costs estimated from average cost variance for biomass-based methanol and ethylene.
[4] Low and high emission factors calculated using average variance for CO_2-based methanol and ethylene.

Section 3: Sensitivity analysis

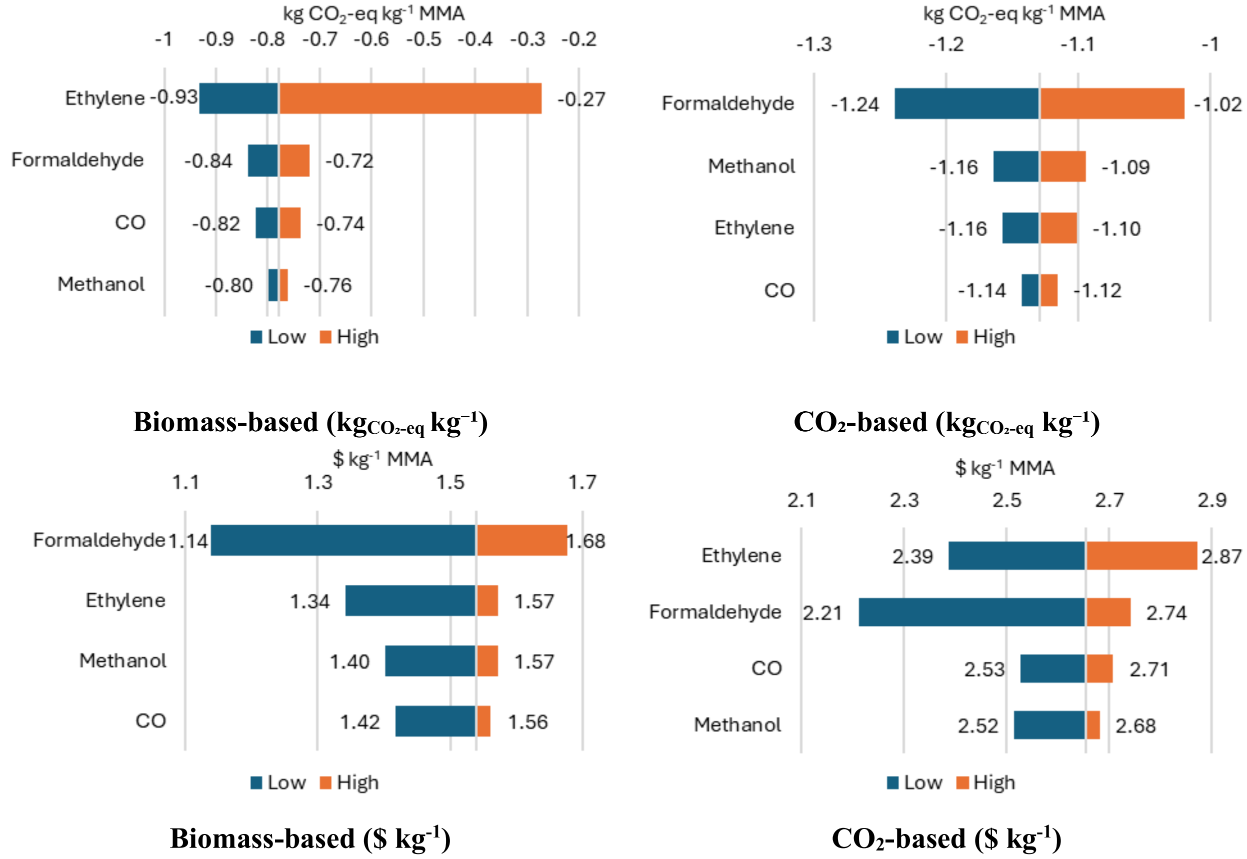

Figure S1: Single-factor sensitivity of the emission factors for MMA (a) biomass-derived and (b) CO_2-derived intermediates and raw-material prices for MMA (c) biomass-derived and (d) CO_2-derived intermediates. Blue bars: low-end cost estimates; orange bars: high-end estimates

Section 4: Technology readiness level

Biomass and CO_2 based ethylene, methanol, and CO are all at different TRLs, the current TRL of these are briefly discussed below. Formaldehyde is not discussed as it is produced from methanol oxidation and is therefore dependant on the TRL of renewable methanol.

1. Ethylene

Bio-based ethylene is currently produced from sugarcane-based bioethanol at a capacity of 260 kt/year (36). Corn-based ethanol in the US is available on a large scale with 16,219 million gallons produced in 2024 (37). However, despite ethanol dehydration to ethylene being a commercial reality there is no current corn-based ethanol to ethylene facilities. As such, corn-based bio-ethylene is estimated to be TRL 7-8 as the individual steps are proven to commercial level, but there is no fully integrated facility.

No operational CO_2 to ethylene facilities currently exist however, research is being undertaken to improve performance (38) and project Air2Chem is hoping to develop economical ethylene from DAC using renewable wind electricity (39). It is estimated this technology is currently at proof-of-concept stage and therefore TRL 2-3.

2. Methanol

Enerkem has produced bio-based methanol from municipal solid waste at its Edmonton site since 2017 (40). Furthermore, Enerkem are constructing a new bio-methanol facility processing >200tn non-recyclable residual material and biomass residues in Québec (41). As such, biomass derived syngas to methanol is estimated to be at TRL 7-8. CO_2 based methanol is currently at demonstration scale (TRL 4-6).

Greenlyte recently secured funding for a 1 kt yr^{-1} DAC to e-methanol facility in Germany (42) and 4 kt yr^{-1} plant operates in Iceland using CO_2 sourced from a geothermal power plant in Iceland (43). Similarly, the Haru Oni demonstration in Chile uses wind energy to produce CO_2 based methanol from DAC and water electrolysis. The plant has a 350 tn yr^{-1} capacity and has been operation since December 2022 (44).

3. CO

Syngas production from biomass is currently at TRL 8 according to the 2021 IEA status report on thermal gasification. The report identifies several syngas production facilities operating at this TRL across the UK and USA (45). Separation to produce pure CO is commercial for fossil-derived syngas so the same infrastructure could be utilised for bio-derived.

It is estimated that CO from DAC is currently at TRL 7-8 with Haldor Topsoe commissioning a 12 Nm^3 hr^{-1} CO plant in 2016 (46) and their technology is purchasable for small scale CO_2 upgrading (47).

4. Summary

In summary, the state of renewable intermediates for MMA from biomass resources is at stages 7-9 whilst CO_2 based intermediates from DAC ranges from TRL 2-8 with CO at stages 7-8 and both ethylene and methanol at earlier stages. The earlier TRL of the CO_2 technology is a likely cause of the higher price estimate for these chemicals and reinforces the suggestion that biomass derived chemical intermediates are a more feasible short-term feedstock for renewable MMA production.

References

1. Adeoye, F., Passarini, J., De Maron, T., Tabanelli, F., Cavani, F. and Cespi, D. (2023) 'Methyl methacrylate production processes: A comparative analysis of alternatives using the life-cycle assessment methodology', ACS Sustainable Chemistry & Engineering, 11 (49), pp. 17355–17370.
2. Parvatker & Eckelman (2020) Simulation-Based Estimates of Life Cycle Inventory Gate-to-Gate Process Energy Use for 151 Organic Chemical Syntheses, ACS Sustainable Chemistry & Engineering, 8, 23, 8519–8536. https://doi.org/10.1021/acssuschemeng.0c00439
3. Moraru, M,D., Bildea, C.S., Kiss, A.A., (2021), Novel Eco-Efficient Process for Methyl Methacrylate Production. Industrial & Engineering Chemistry Research, 60, 3, 1290–1301. https://doi.org/10.1021/acs.iecr.0c04273
4. Argonne National Laboratory (2022) The GREET® model (Version 2022). Available at: https://greet.es.anl.gov (Accessed: 22 May 2025).
5. Han, J., Sen, M.S., Alonso, D.M., Dumesic, J.A. and Maravelias, C.T. (2014) 'Simultaneous catalytic conversion of hemicellulose and cellulose from lignocellulosic biomass to liquid transportation fuels', Green Chemistry, 16 (1). https://doi.org/10.1039/C3GC41511B
6. Business Analytiq (2025a) Silica-sand price index. Available at: https://businessanalytiq.com/procurementanalytics/index/silica-price-index/ (Accessed: 6 May 2025).
7. Statista (2025) Average import price of zirconium in the United States, 2018–2023. Available at: https://www.statista.com/statistics/1318970/average-price-of-zirconium/ (Accessed: 6 May 2025).
8. SMM (2025) Cesium-carbonate (Cs_2CO_3 ≥ 99 %) price (USD kg^{-1}). Available at: https://www.metal.com/Other-Minor-Metals/202012250002 (Accessed: 6 May 2025).
9. Benavides, P.T., Gracida-Alvarez, U.R., Richa, K., Port, J. and Hawkins, T.R. (2025) 'Cradle-to-gate greenhouse-gas emissions of the production of ethylene from U.S. corn ethanol and comparison with fossil-derived ethylene', Bioresource Technology, 430. https://doi.org/10.1016/j.biortech.2025.132565
10. Intratec. *Ethylene Price - Current & Forecasts*. Intratec.us. Updated 6 April 2026. Accessed 13 April 2026.
11. Nicholson, S. R.; Rorrer, N. A.; Uekert, T.; Avery, G.; Carpenter, A. C.; Beckham, G. T. Manufacturing Energy and Greenhouse Gas Emissions Associated with United States Consumption of Organic Petrochemicals. *ACS Sustain. Chem. Eng.* 2023, *11* (6), 2198–2208. https://doi.org/10.1021/acssuschemeng.2c05417
12. PriceWatch. *Methanol Price Trend and Forecast*; PriceWatch. Accessed April 13, 2026.
13. BusinessAnalytiq. *Methanol Price Index*; BusinessAnalytiq. Accessed April 13, 2026.
14. PriceWatch. *Formaldehyde Price Trend and Forecast*; PriceWatch. Accessed April 13, 2026.
15. Roh, K.; Lee, S.; Kwon, Y.; An, J. Life Cycle Assessment of Fossil-Fuel based Carbon Monoxide Production: Comparison of Steam Methane Reforming and Partial Oxidation. *Korean J. Life Cycle Assess.* 2024, *25* (2), 47–54. https://doi.org/10.62765/kjlca.2024.25.2.47
16. StatsNex Market Insights. *Carbon Monoxide Market Size ($6.8 Billion) by 2032*; StatsNex Market Insights. Accessed April 13, 2026.
17. Yang, M., Tian, X. and You, F. (2018) 'Manufacturing ethylene from wet shale gas and biomass: Comparative techno-economic analysis and environmental life-cycle assessment', Industrial & Engineering Chemistry Research, 57 (17). https://doi.org/10.1021/acs.iecr.7b03731
18. McKechnie, J., Pourbafrani, M., Saville, B.A. and MacLean, H.L. (2015) 'Environmental and financial implications of ethanol as a bio-ethylene feedstock versus a transportation fuel', Environmental Research Letters, 10 (12). https://doi.org/10.1088/1748-9326/10/12/124018

19. Schill, S.R. (2013) ‘Intratec report evaluates ethanol-to-ethylene economics’, Ethanol Producer Magazine, 19 August. Available at: https://ethanolproducer.com/articles/intratec-report-evaluates-ethanol-to-ethylene-economics-10023 (Accessed: 29 April 2025).
20. Hamelinck, C. and Bunse, M. (2022) Carbon footprint of methanol. Amsterdam: Studio Gear Up. Available at: https://www.methanol.org/wp-content/uploads/2022/01/Carbon-Footprint-of-Methanol-studio-Gear-Up-Full-Presentation.pdf (Accessed: 30 April 2025).
21. IRENA (2021) Innovation outlook: Renewable methanol. Abu Dhabi: IRENA. Available at: https://www.irena.org/-/media/Files/IRENA/Agency/Publication/2021/Jan/IRENA_Innovation_Renewable_Methanol_2021.pdf (Accessed: 29 April 2025).
22. Wernet, G., Bauer, C., Steubing, B., Reinhard, J., Moreno-Ruiz, E., & Weidema, B. P. (2016). The ecoinvent database version 3 (part I): overview and methodology. International Journal of Life Cycle Assessment, 21(9), 1218–1230. https://doi.org/10.1007/s11367-016-1087-8
23. Intratec Solutions LLC (2023, 4 October). *Intratec Water & Utility Costs* (annual subscription database). Intratec Solutions LLC.
24. Bachman, M., Völker, S., Kleinekorte, J. and Bardow, A. (2023) ‘Syngas from what? Comparative life-cycle assessment for syngas production from biomass, CO_2 and steel-mill off-gases’, ACS Sustainable Chemistry & Engineering, 11 (14). https://doi.org/10.1021/acssuschemeng.2c05390
25. Air Liquide (2023) Technology handbook. Available at: https://engineering.airliquide.com/sites/engineering/files/2022-09/technohandbook11oct.pdf (Accessed: 22 May 2025).
26. Hinchcliffe, A.B. (1991) The separation of hydrogen and carbon monoxide using polymer membranes. PhD thesis. Aston University. https://doi.org/10.48780/publications.aston.ac.uk.00009756
27. Rodgers, S., Meng, F., Poulston, S., Conradie, A. and McKechnie, J. (2022) ‘Renewable butadiene: A case for hybrid processing via bio- and chemo-catalysis’, Journal of Cleaner Production, 364, 132614. https://doi.org/10.1016/j.jclepro.2022.132614
28. Kim, S. and Benavides, P. (2024) ‘Comparative life-cycle analysis of ethylene production from electrocatalytic CO_2 reduction’, Journal of Cleaner Production, 449. https://doi.org/10.1016/j.jclepro.2024.141348
29. Leonzio, G., Chachuat, B. and Shah, N. (2023) ‘Towards ethylene production from carbon dioxide: Economic and global-warming-potential assessment’, Sustainable Production and Consumption, 43, pp. 101–115.
30. IEA (2022) Direct Air Capture 2022. Paris: International Energy Agency. Available at: IEA executive summary page (Accessed: 3 March 2026). Badger, N., Boylu, R., Ilojianya, V., Erguvan, M. and Amini, S. (2024) ‘A cradle-to-gate life-cycle assessment of green methanol production using direct air capture’, Energy Advances. https://doi.org/10.1039/D4YA00316K
31. Leonzio, G., Hankin, A. and Shah, N. (2024) ‘CO_2 electrochemical reduction: A state-of-the-art review with economic and environmental analyses’, Chemical Engineering Research and Design, 208. https://doi.org/10.1016/j.cherd.2024.07.014
32. Cameli, F., Delikonstantis, E., Kourou, A., Rosa, V., Van Geem, K.M. and Stedanidis, G.D. (2024) ‘Conceptual process design and techno-economic analysis of an e-methanol plant with direct-air-captured CO_2 and electrolytic H_2’, Energy & Fuels, 38 (4), pp. 3251–3261. https://doi.org/10.1021/acs.energyfuels.3c04147
33. Rosental, M.R., Fröhlich, T.F. and Liebich, A.L. (2020) ‘Life Cycle Assessment of Carbon Capture and Utilization for the Production of Large Volume Organic Chemicals’, *Frontiers in Climate*, 2. doi: 10.3389/fclim.2020.586199
34. Huang, Z., Grim, R.G., Schidle, J.A. and Tao, L. (2021) ‘The economic outlook for converting CO_2 and electrons to molecules’, Energy & Environmental Science, 14 (7), pp. e1–e14.

35. Nabil, S.K., McCoy, S. and Kibria, M.G. (2021) ‘Comparative life-cycle assessment of electrochemical upgrading of CO_2 to fuels and feedstocks’, Green Chemistry, 23 (24). https://doi.org/10.1039/D0GC02831B
36. Braskem (2023) ‘Braskem expands its biopolymer production by 30 % following an investment of US$ 87 million’. Press release [Online]. Available at: https://www.braskem.com.br/europe/news-detail/braskem-expands-its-biopolymer-production-by-30-following-an-investment-of-us-87-million (Accessed: 9 May 2025).
37. RFA (2024), Annual Ethanol Production - U.S. and World Ethanol Production. Available at: https://ethanolrfa.org/markets-and-statistics/annual-ethanol-production (Accessed: 29 April 2025).
38. Ni, W., Chen, H., Tang, N. et al. (2024) ‘High-purity ethylene production via indirect carbon-dioxide electrochemical reduction’, Nature Communications, 15, 6078. https://doi.org/10.1038/s41467-024-50522-7
39. Fraunhofer (2024) ‘Electrolysis meets “Direct Air Capture”’. Press release [Online]. Available at: https://www.umsicht.fraunhofer.de/en/press-media/press-releases/2024/direct-air-capture-electrolysis.html (Accessed: 9 May 2025).
40. EDI (2023) ‘World’s first municipal waste-to-biofuels plant opens in Edmonton’. Available at: https://www.ediweekly.com/worlds-first-municipal-waste-biofuels-plant-opens-edmonton/ (Accessed: 9 May 2025).
41. Recyclagecarbone, 2025. Biofuel produced through waste recovery. Available at: https://recyclagecarbone.com/que-faisons-nous/ (Accessed: 9 May 2025).
42. Greenlyte (2025) Greenlyte secures multi-million-euro funding to reach commercial inflection point by building its FOAK-1 facility. Available at: https://www.greenlyte.tech/milestones/greenlyte-secures-multi-million-euro-funding-to-reach-commercial-inflection-point-by-building-its-foak-1-facility (Accessed: 9 May 2025).
43. Carbon Recycling International (n.d.) Renewable methanol plant: First production of fuel from CO_2 at industrial scale. Available at: https://carbonrecycling.com/projects/george-olah (Accessed: 9 May 2025).
44. HIF (2023) Haru Oni plant: ISCC Plus certification experience of e-fuels. Available at: https://www.iscc-system.org/wp-content/uploads/2023/08/3.3_Pedinian_TC-LATAM_-HIF-Global-Haru-Oni-ISCC-PLUS-certification.pdf (Accessed: 9 May 2025).
45. IEA (2021) Status report on thermal gasification of biomass and waste 2021. Paris: IEA Bioenergy, Task 33. Available at: https://www.ieabioenergy.com/wp-content/uploads/2022/03/FS-op-final.pdf (Accessed: 22 May 2025).
46. Mittal, C., Hadsbjerg, C. and Blennow, P. (n.d.) ‘Small-scale CO from CO_2 using electrolysis’, CEW Features. Available at: https://www.topsoe.com/hubfs/2115834/CAMPAIGNS/eCOs/Haldor%20Topsoe%20India%20article.pdf (Accessed: 22 May 2025).
47. TOPSOE (2025) CO production decarbonised. Available at: https://www.topsoe.com/processes/carbon-monoxide (Accessed: 9 May 2025).
